# miR-369 inhibits Liver Cancer progression by targeting ZEB1 pathway and predicts the prognosis of HCC patients

**DOI:** 10.7150/jca.54759

**Published:** 2021-03-19

**Authors:** Yuwei Dong, Fuxia Li, Junjun Wang, Jiangfeng Hu, Zhenghong Li, Yubei Gu, Yun Feng

**Affiliations:** 1 Department of Gastroenterology, Shanghai General Hospital, School of Medicine, Shanghai Jiao Tong University. Shanghai, 200080, China.; 2 Department of General Surgery, Cao County People's Hospital, Heze, Shandong province, 274400, China.; 3 Department of Gastroenterology, Rui Jin Hospital, School of Medicine, Shanghai Jiao Tong University. Shanghai, 200025, China.

**Keywords:** hepatocellular carcinoma, miR-369, ZEB1, prognosis, progression

## Abstract

Increasing evidences show that microRNAs (miRNAs) are involved in the regulation of tumorigenesis, progression, recurrence and drug resistance of hepatocellular carcinoma (HCC). miR-369 works as a tumor suppressor in both lung cancer and thyroid cancer. However, the potential biological function of miR-369 in HCC is unknown. Herein, we for first found that miR-369 expression was downregulated in HCC tissues and predicted the poor prognosis of HCC patients. Forced miR-369 expression inhibited the proliferation and metastasis of HCC cells *in vitro* and *in vivo*. Mechanically, bioinformatics and luciferase reporter analysis identified Zinc finger E-box binding homeobox 1 (ZEB1) as a direct target of miR-369 in HCC cells. miR-369 overexpressing downregulated the ZEB1 mRNA and protein expression in HCC cells. miR-369 expression was negatively associated with ZEB1 expression in human HCC tissues. More importantly, the ZEB1 siRNA diminished the discrepancy of growth and metastasis capacity between miR-369 overexpression HCC cells and control cells.

## Introduction

Hepatocellular carcinoma (HCC) is one of the most lethal malignancy in the world. The prognosis of HCC is extremely poor with a five-year survival rate of less than 30% 1, 2. The early symptoms of liver cancer are not obvious. Most patients are diagnosed at advanced stage and lost the best operative chance. Moreover, the metastasis and recurrence rate of HCC remains very high 3, 4. For these inoperative patients, TACE and targeted drugs are the last option. However, most patients are not sensitive to TACE or targeted drugs 5-7. Therefore, a better understanding of the molecular mechanisms of HCC is indispensable for discovering novel and effective therapeutic approaches for the treatment and identifying new biomarkers that can reflect therapeutic responses.

MicroRNAs (miRNAs) are small and endogenous noncoding RNA that regulate gene expression by interacting with the 3′‐non‐translation region (3′‐untranslated region [3′‐UTR]) of the target gene messenger RNA (mRNA) to inhibit translation or promote mRNA degradation 8-10. miRNA have been demonstrated to be involved in embryonic development, organogenesis, and also multiple physiological processes including cell signaling, metabolic processes, cell cycle control, immune responses, and even cognitive function 11-14. miRNA‐369 (miR‐369) was reported to inhibit papillary thyroid cancer proliferation by downregulating TSPAN13 expression 15. Attenuation of deregulated miR-369 expression sensitizes non-small cell lung cancer cells to cisplatin via modulation of the nucleotide sugar transporter SLC35F5 16. In addition, miR-369 suppresses cell migration and proliferation by targeting SOX4 in Hirschsprung's disease 17. However, the role of miR‐369 in HCC and its potential mechanism are still unclear.

In the present study, we for first found that the expression of miR-369 was downregulated in liver fibrosis and liver cancer tissues. The analysis of patient cohort demonstrated that miR-369 predicted the poor prognosis of HCC patients. Biological function study demonstrated that miR-369 overexpression inhibited the proliferation, metastasis of HCC cells. Further mechanism study reveals that miR-369 inhibited HCC cells proliferation and metastasis by directly targeting ZEB1 pathway.

## Materials and Methods

### Human tissue samples

HCC samples were collected from patients who underwent the resection of their primary HCC in the Shanghai General Hospital. A total of 120 patients were followed for 5 years, and recurrence free survival (DFS) and OS analyses were performed using the Kaplan-Meier method. OS was defined as the interval between the dates of surgery and death. The recurrence was defined as the interval between the dates of surgery and recurrence; if recurrence was not diagnosed, then patients were censored on the date of death or on the last follow-up. Detailed clinicopathological features of the patients in Supplementary Table 1. The control liver tissues (n=10) used for the analysis were the distal para-haemangioma tissues without any abnormality from patients who underwent surgical resection for hepatic haemangioma at Shanghai General Hospital. The fibrotic liver tissues (n=20) were from patients with hepatic fibrosis. HCC tissues and their normal controls tissues were obtained from HCC patients who under surgical remove in Shanghai General Hospital. The liver tissues were snap frozen and the RNA was extracted for further analysis. The patients' informed consent was also obtained, and all procedures were approved by the ethical committee of Shanghai General Hospital.

### Cell lines and cell culture

HCC cell lines HCCLM3 and Huh7 cells were purchased from Chinese Academy of Sciences, Shanghai, China. The HCC cells were cultured in Eagle's Minimum Essential Medium supplemented with 10 % fetal calf serum (FCS; Invitrogen, Carlsbad, CA, USA). LV3-has-miR-369 mimic virus and LV3-has-miR-369 sponge virus were purchased from Shanghai GenePharma (Shanghai, China). HCCLM3 and Huh7 cells were dissociated with 0.5% trypsin and seeded into six-well plates. Then the cells were infected with miR-369 mimic virus or miR-369 sponge virus and their control virus. The stable infectants were screening by using puromycin described as before 18.

HCCLM3 miR-369 mimic or Huh7 miR-369 mimic and their control cells were seeded into a six-well plate until they reached 60-70% confluence. Transfection of ZEB1 siRNA or its negative control siRNA was performed in each well in the absence of serum with siRNA transfection reagent according to the manufacturer's instructions (Polyplus, Illkirch, France). The sequence of si-ZEB1 is as follows: 5'- CCUCUCUGAAAGAACACAUUA -3'. Then the above cells were subjected to CCK8 assay, transwell assay and matrigel invasion chamber assay.

### Animal models

Male C57BL/6 mice aged 6-8 weeks were intraperitoneally injected with carbon tetrachloride (CCl4) (0.25 mL/kg body weight) or vehicle (olive oil) three times per week for 8 weeks to induce fibrosis and were sacrificed 4 days after the last injection (n=8) 19. The mice subjected to bile duct ligation (BDL) were anaesthetized with ketamine and xylazine followed by midline laparotomy (n=8) 19. The common bile duct was ligated two times with 6-0 silk sutures and cut through between the ligations. Sham-operated mice were subjected to laparotomy without BDL. The mice that received BDL or the sham operation were sacrificed 15 days later. The mouse livers were collected for determined miR-369 expression.

For xenograft formation assay, HCCLM3 miR-369 mimic and its control cells (2×10^6^) were injected subcutaneously into nude mice (n=6). Nude mice were sacrificed six weeks post inoculation and tumors were collected and examined.

For tail vein lung metastasis assay, HCCLM3 miR-369 mimic and its control cells (2×10^6^) were intravenously injected into male nude mice through the tail vein (Chinese Science Academy, Shanghai, China) (n=7). After three months, lung metastasis was measured. All animal procedures were conducted with the approval of the Shanghai General Hospital and the School of Medicine, Shanghai Jiao Tong University.

### Cell proliferation assays

For CCK8 assay, HCCLM3 miR-369 mimic/sponge or Huh7 cells miR-369 mimic/sponge and their control cells were seeded in 96-well plates (3×10^3^ cells per well). ATP activity was measured using a Cell Counting Kit-8 at indicated time points. ATP activity was measured using a Cell Counting Kit-8 at indicated time points. The procedure was as follows: The cell suspension (100 μl/well) was inoculated in a 96-well plate, and the plate was pre-incubated in a humidified incubator at 37 °C for 1 hour. This was followed by the addition of 10 μl of the CCK-8 solution to each well of the plate, and incubation of the plate for 1 h in the incubator. Finally, the absorbance was measured at 450 nm using a microplate reader (Synergy H1; BioTek Instruments, Inc., Winooski, VT, USA).

For colony formation assay, HCCLM3 miR-369 mimic/sponge or Huh7 cells miR-369 mimic/sponge and their control cells were cultured in 12-well plates (3×10^3^ cells/well). The cells were incubated at 37 °C for 7 days and then fixed with with 10% neutral formalin for over 4 hours. The cells were dyed with crystal violet (Beyotime, Haimen, China). The cells were photographed under a microscope (Olympus, Tokyo, Japan).

For cell EdU immunofluorescence staining, HCCLM3 miR-369 mimic or Huh7 cells miR-369 mimic and their control cells were seeded into 96-well plates and performed using the EdU Kit (RiboBio) at 48 hours. The results were quantified with a Zeiss axiophot photomicroscope (Carl Zeiss) and Image-Pro plus 6.0 software.

### Cell metastasis assays

For cell migration experiments, 2×10^5^ HCCLM3 miR-369 mimic or Huh7 cells miR-369 mimic and their control cells were seeded into the upper chamber of a polycarbonate transwell in serum-free DMEM medium. The lower chamber was added with DMEM medium containing 20% FBS as chemoattractant. The cells were incubating for 16 hours and the chamber was fixed with 10% neutral formalin for more than 4 hours. The cells were dyed with crystal violet (Beyotime). The cells were then counted under a microscope (Olympus) and the cell number is expressed as the average number of the cells in each field.

For cell invasion experiments, 2×10^5^ HCCLM3 miR-369 mimic/sponge or Huh7 cells miR-369 mimic/sponge and their control cells were seeded into the upper chamber of a polycarbonate transwell in serum-free DMEM medium. The lower chamber was added with DMEM medium containing 20% FBS as chemoattractant. The cells were incubating for 36 hours and the chamber was fixed with 10% neutral formalin for more than 4 hours. The cells were dyed with crystal violet (Beyotime). The cells were then counted under a microscope (Olympus) and the cell number is expressed as the average number of the cells in each field.

### Real-time PCR

For detection of mature miR-369, total RNA was subjected to reverse transcription using a TaqMan MicroRNA Reverse Transcription Kit (Applied Biosystems). qRT-PCR analysis of miR-552 expression was carried out using TaqMan MicroRNA assay kits (Applied Biosystems). Results were normalized to U6 snRNA using the comparative threshold cycle (Ct) method. The miR-369 primer sequences were forward: 5' AAUAAUACAUGGUUGAUCUUU 3', U6 primer sequences were forward: 5' ATTGGAACGATACAGAGAAGATT 3'.

The total cells RNA was extracted by using Trizol reagent (Invitrogen, 15596-018). Total cDNAs were synthesized by ThermoScript TM RT-PCR system (Invitrogen, 11146-057). The total mRNA amount presented in the cells was measured by RT-PCR using the ABI PRISM 7300 sequence detector (Applied Biosystems). The ZEB1 primer sequences were forward: 5' GATGACCTGCCAACAGACCA 3', reverse: 5' CTGTGTCATCCTCCCAGCAG 3'. The β-actin was used as reference for relative expression calculation and its primer sequences were forward: 5' GGCCCAGAATGCAGTTCGCCTT 3', reverse: 5' AATGGCACCCTGCTCACGCA 3'.

### Western blotting assays

Thirty micrograms of proteins were subjected to sodium dodecyl sulfate polyacrylamide gel electrophoresis and then transferred to the nitrocellulose membrane. The membrane was blocked with 5% non-fat milk and incubated with the primary antibody for 1.5 hours. The protein band, specifically bound to the primary antibody, was detected using an IRDye 800CW-conjugated secondary antibody and LI-COR imaging system (LI-COR Biosciences). The primary antibodies were ZEB1 (1:1000; ab203829, Abcam), GAPDH (1:5000; ab181602, Abcam) and β-actin (1:5000; 60008-1-Ig, Proteintech).

### Luciferase reporter assays

The wide type ZEB1 3'-untranslated region (UTR) containing miR-369 targeting sequence (GUAUUAUU) and the mutated type (AGCGAGUU) was amplified and cloned into the luciferase reporter plasmid pGL4.13 vector (Promega, Madison, WI). All the constructs were verified by sequencing. Briefly, laryngocarcinoma cells were co-transfected with miR-369 mimic or miR-control and pMIR-reporter luciferase vector containing a specific sequence of wild-type or mutant ZEB1 fragment, using siRNA transfection (Invitrogen, NY, USA). Cells were collected and lysed for luciferase detection 48 hours after transfection. The relative luciferase activity was normalized against to the Renilla luciferase activity.

### Statistical analysis

All statistical analyses were performed using GraphPad Prism (GraphPad Software, Inc. La Jolla, USA). Statistical analysis was carried out using t test or Bonferroni Multiple Comparisons Test: *p<0.05. A p value of less than 0.05 was considered statistically significant.

## Results

### miR-369 expression is downregulated in liver fibrosis and liver cancer tissues

**Figure 1:**
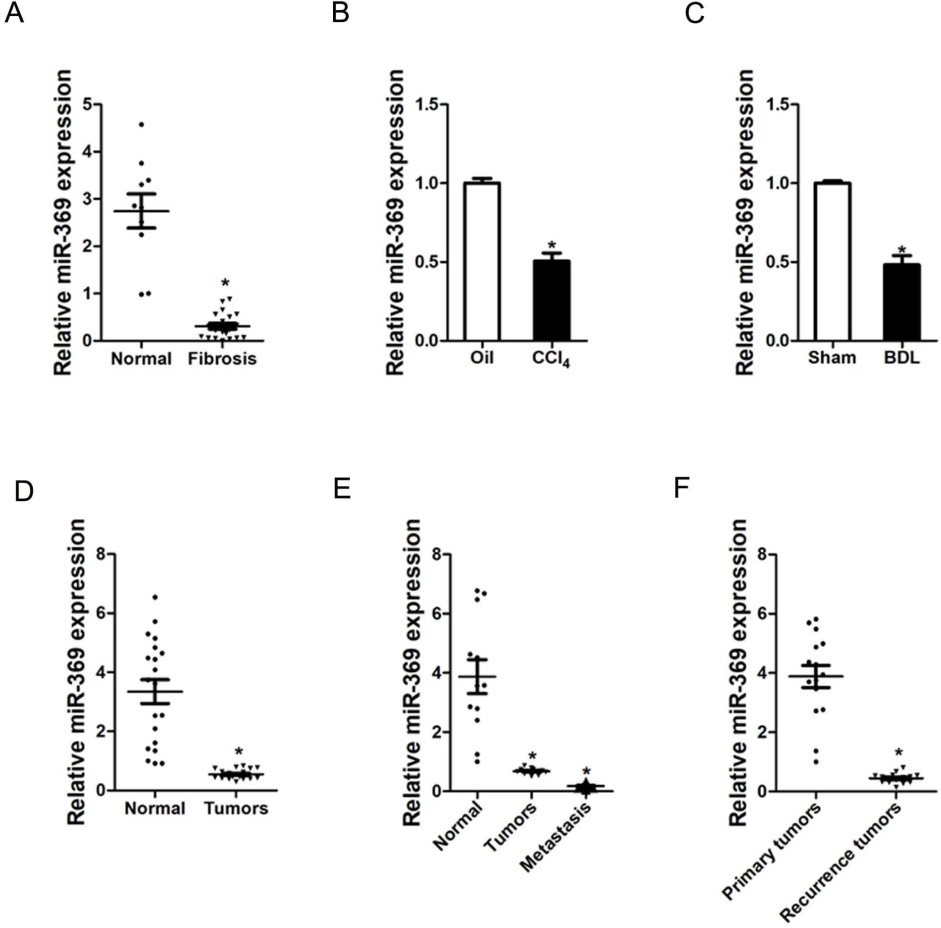
** Expression of miR-369 is downregulated in liver fibrosis and liver cancer tissues. A.** Real-time PCR analysis of miR-369 in patient fibrotic tissues (n=20) or normal tissues (n=10) from liver (p<0.05). **B.** Real-time PCR analysis of miR-369 in CCL4-induced fibrosis mice (n=8) (p<0.05). **C.** Real-time PCR analysis of miR-369 in BDL-induced fibrosis mice (n=8) (p<0.05). **D.** The expression of miR-369 in human liver cancer patients' tissues and their corresponding adjacent normal tissues (n=20) was checked by real-time PCR analysis (p<0.05). **E.** The expression of miR-369 in adjacent normal tissues, liver cancer tissues and their corresponding metastasis tissues (n=12) was checked by real-time PCR analysis (p<0.05). **F.** The expression of miR-369 in primary liver cancer tissues and recurrence liver cancer tissues was investigated via real-time PCR analysis (n=15) (p<0.05) (Data are presented as mean ± SEM).

To explore the role of miR-369 in liver fibrosis and liver cancer, we first examined the expression of miR-369 in human liver tissues. As shown in Figure 1A, the expression of miR-369 was significantly decreased in fibrotic livers from patients compared with healthy individuals. Moreover, miR‐369 expression level was obtained with significantly downregulation in the CCl4-induced or BDL-induced murine liver fibrosis (Fig. 1B & C). Next, we found that miR-369 level was dramatically reduced in HCC tissues compared with their normal controls (Fig. 1D). HCC carries a high risk of portal vein metastasis, and metastatic foci in portal vein markedly deteriorate hepatic function and serve as a prognostic factor of poor prognosis of patient 20. Strikingly, miR-369 expression was significantly downregulated in metastatic foci compared with the matched primary HCCs or peri-tumor normal tissues, which indicates the potential role of miR-369 in HCC metastasis (Fig. 1E). Furthermore, we also found that miR-369 expression in recurrence HCC tissues was significantly lower than the primary HCC tissues (Fig. 1E). Collectively, the above results demonstrated that miR-369 might play an important function in HCC progression.

### miR-369 predicts the poor prognosis of HCC patients

**Figure 2:**
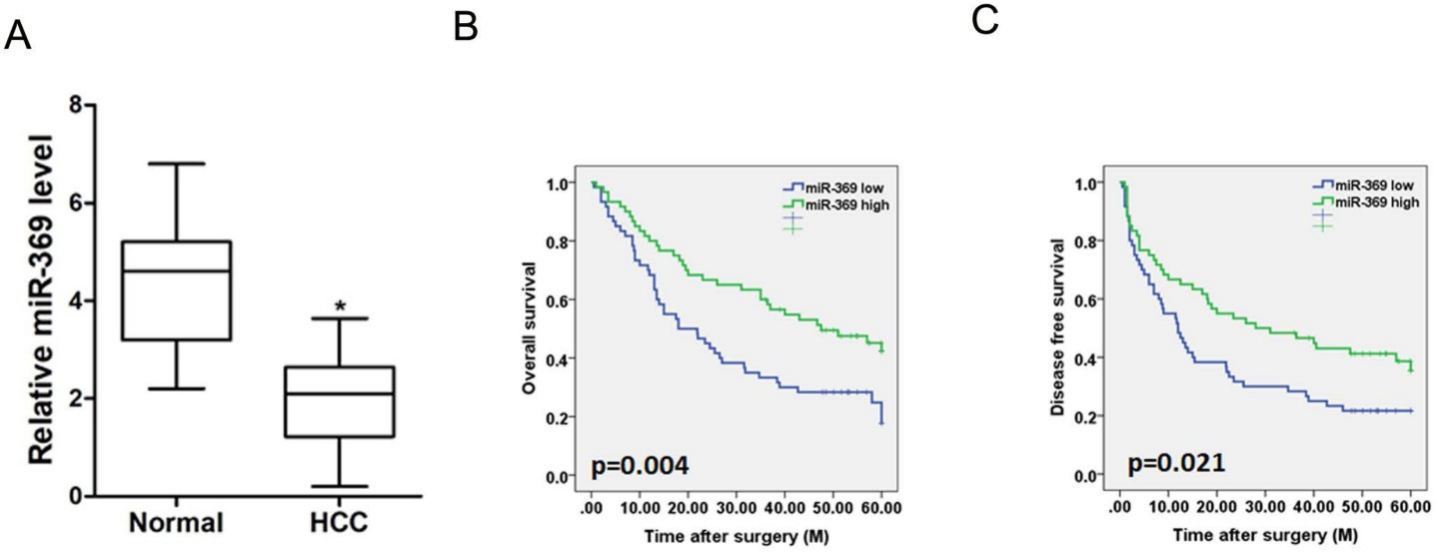
** Low expression of miR-369 predicts the poor prognosis of HCC patients. A.** Real-time PCR analysis of miR-369 in HCCs and peri-tumor normal tissues from 120 patients (p<0.05). **B & C.** Real-time PCR and scoring of miR-369 expression were performed in 120 human HCC samples. Overall survival time and disease-free survival after surgery of the patients were compared between the ''miR-369 low'' (n = 60) and ''miR-369 high'' (n = 60) groups, p <0.05.

To investigate the clinical significance of miR-369, we checked miR-369 expression in a total of 120 HCC tissues. The average level of miR-3619 was lower in HCC tumors than adjacent non-tumor tissues determined by real-time PCR (Fig. 2A). We then analyzed the correlation between miR-369 expression and the clinicopathological characteristics in 120 HCC patients. As summarized in Supplementary Table 1, low levels of miR-369 expression were significantly correlated with increased HBsAg (p<0.05), AFP (p<0.05) tumor size (p<0.05), portal vein tumor thrombus (p<0.05) and BCLC stage (p<0.05). Moreover, Kaplan-Meier survival analysis with the log-rank test revealed that patients with lower miR-369 levels exhibited worse overall survival and shorter time to recurrence (Fig. 2B & C).

### miR-369 inhibits HCC cells proliferation

**Figure 3:**
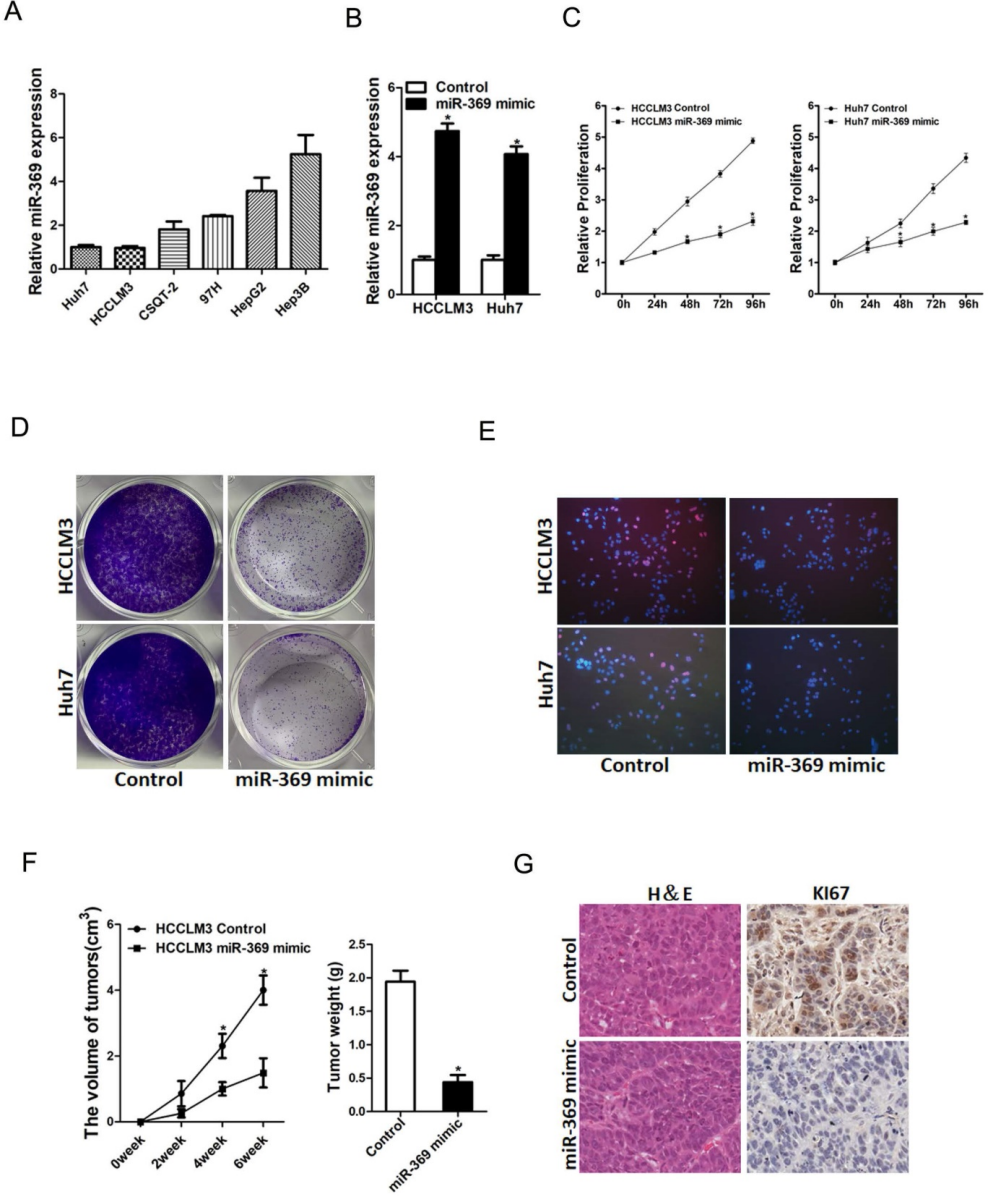
** Overexpression of miR-369 inhibits HCC cells proliferation. A.** The expression of miR-369 in HCC cell lines was checked by real-time PCR assay. **B.** The overexpression effect of miR-369 in HCCLM3 and Huh7 cells was checked by real-time PCR assay. **C.** Cell proliferation was measured using CCK-8 assays in HCCLM3 miR-369 mimic or Huh7 miR-369 mimic and their control cells. **D.** Colony formation assays of HCCLM3 miR-369 mimic or Huh7 miR-369 mimic and their control cells. **E.** Cell proliferation was assessed using EdU immunofluorescence staining (red) in HCCLM3 miR-369 mimic or Huh7 miR-369 mimic and their control cells. Nuclei were stained with Hoechst 33342 (blue). **F.** HCCLM3 miR-369 mimic and its control cells were implanted subcutaneously to induce xenograft tumor in nude mice (n=6). Xenografted tumor growth was monitored and tumor weight was measured 6 weeks later. **G.** Xenograft tumors were excised and subjected to IHC staining.

To further explore the potential biological effect of miR-369 on HCC cells behavior, we checked the expression of miR-369 expression in a great many HCC cell lines and found miR-369 level in HCCLM3 and Huh7 cells was lower than other HCC cell lines (Fig. 3A). So, we chose this two HCC cell lines in our study. HCCLM3 and Huh7 cells were infected with miR-369 mimic virus and the overexpressing effect was determined by real-time PCR (Fig. 3B). HCCLM3 and Huh7 cells were also infected with miR-369 sponge virus and the interference effect was checked by real-time PCR (Fig. S1A). CCK8 assay was used to examine the cell growth; the result showed that miR-369 overexpression inhibited cell growth in HCC cells and miR-369 interference promoted HCC cells growth (Fig. 3C & Fig. S1B). Next, the colony formation assay used to measure cell proliferation, the data showed that miR-369 overexpression HCC cells formed less and smaller colonies compared with control HCC cells (Fig. 3D). Conversely, miR-369 knockdown HCC cells formed much more colonies compared with control cells (Fig. S1C). In addition, 5-ethynyl-2'-deoxyuridine (EdU) staining showed that miR-369 overexpression suppressed HCC cells proliferation (Fig. 3E). More importantly, the *in vivo* experiments also confirmed that miR-369 overexpression inhibited HCC cells growth (Fig. 3F & G). Taken together, the above results showed that miR-369 inhibited HCC cells proliferation.

### miR-369 suppresses HCC cells metastasis

**Figure 4:**
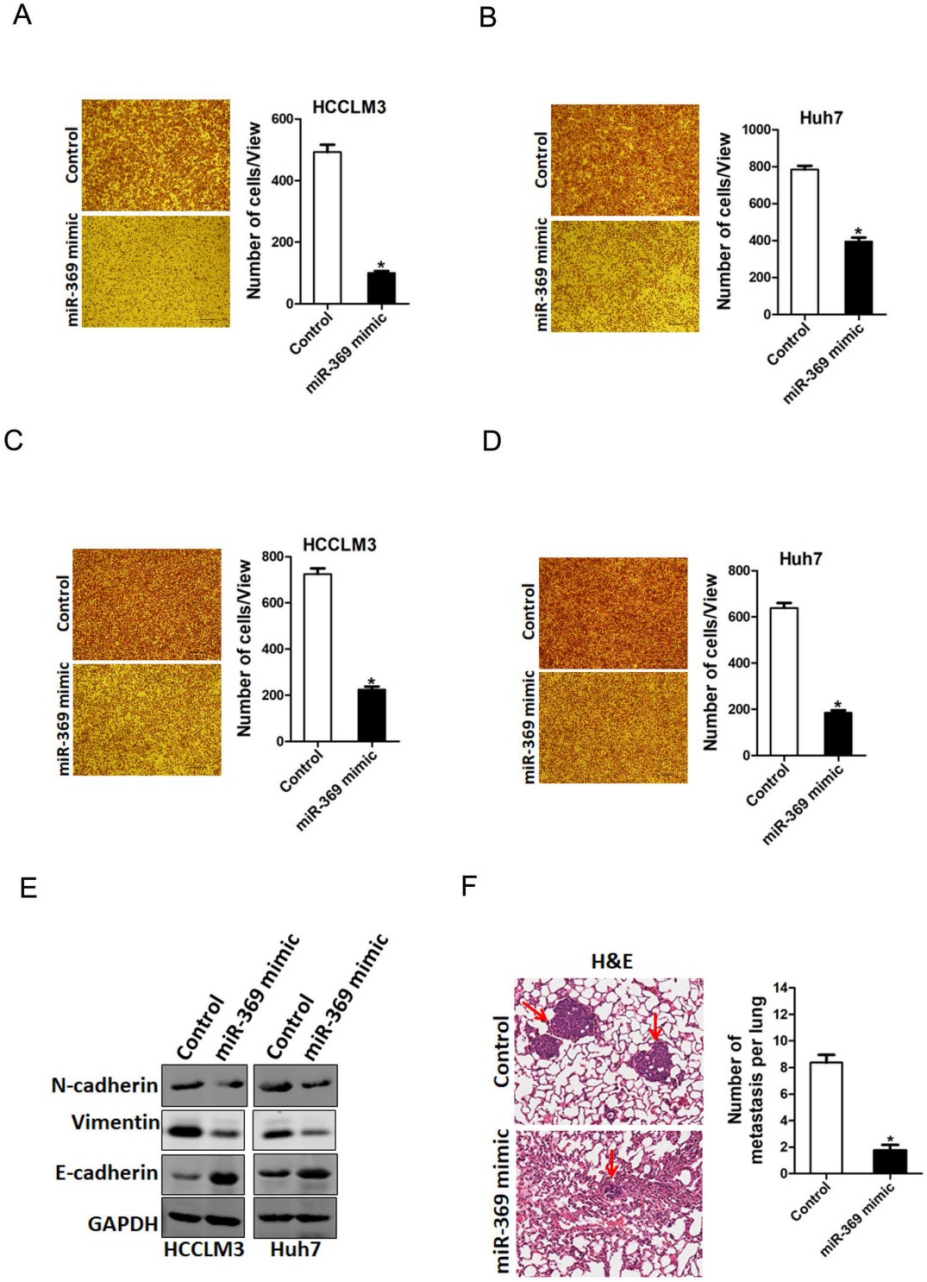
** miR-369 overexpression suppresses HCC cells migration and invasion. A.** The migration ability of HCCLM3 miR-369 mimic and its control cells were performed utilizing polycarbonate membrane inserts in a 24-well plate. **B.** The migration ability of Huh7 miR-369 mimic and its control cells were performed utilizing polycarbonate membrane inserts in a 24-well plate. **C.** The invasive capacity of HCCLM3 miR-369 mimic and its control cells were analyzed using Matrigel-coated Boyden chamber. **D.** The invasive ability of Huh7 miR-369 mimic and its control cells was analyzed using Matrigel-coated Boyden chamber. **E.** H&E staining of nude mice inoculated HCCLM3 miR-369 mimic or control cells via tail vein for 12 weeks. The number of lung metastatic foci in each group (n = 7) were also calculated, p <0.05.

To explore the biological function of miR-369 in HCC cells metastasis, we used transwell assay and matrigel invasion chamber assay. The transwell assay results showed that miR-369 overexpression attenuated the migration ability of HCC cells (Fig. 4A & B). Consistently, matrigel invasion chamber assay revealed that the invasion ability was also impaired in miR-369 overexpression HCC cells (Fig. 4C & D). Conversely, miR-369 knockdown enhanced the migration ability of HCC cells (Fig. S2A & B). Moreover, the EMT protein N-cadherin, vimentin was downregulated and E-cadherin was upregulated in miR-369 overexpression HCC cells (Fig. 4E). Furthermore, miR-369 overexpression HCC cells formed much less metastasis foci in nude mice lung tissues (Fig. 4F). Collectively, our data demonstrated that miR-369 suppresses HCC cells metastasis.

### miR-369 directly targeted ZEB1 to inhibit HCC cells progression

**Figure 5:**
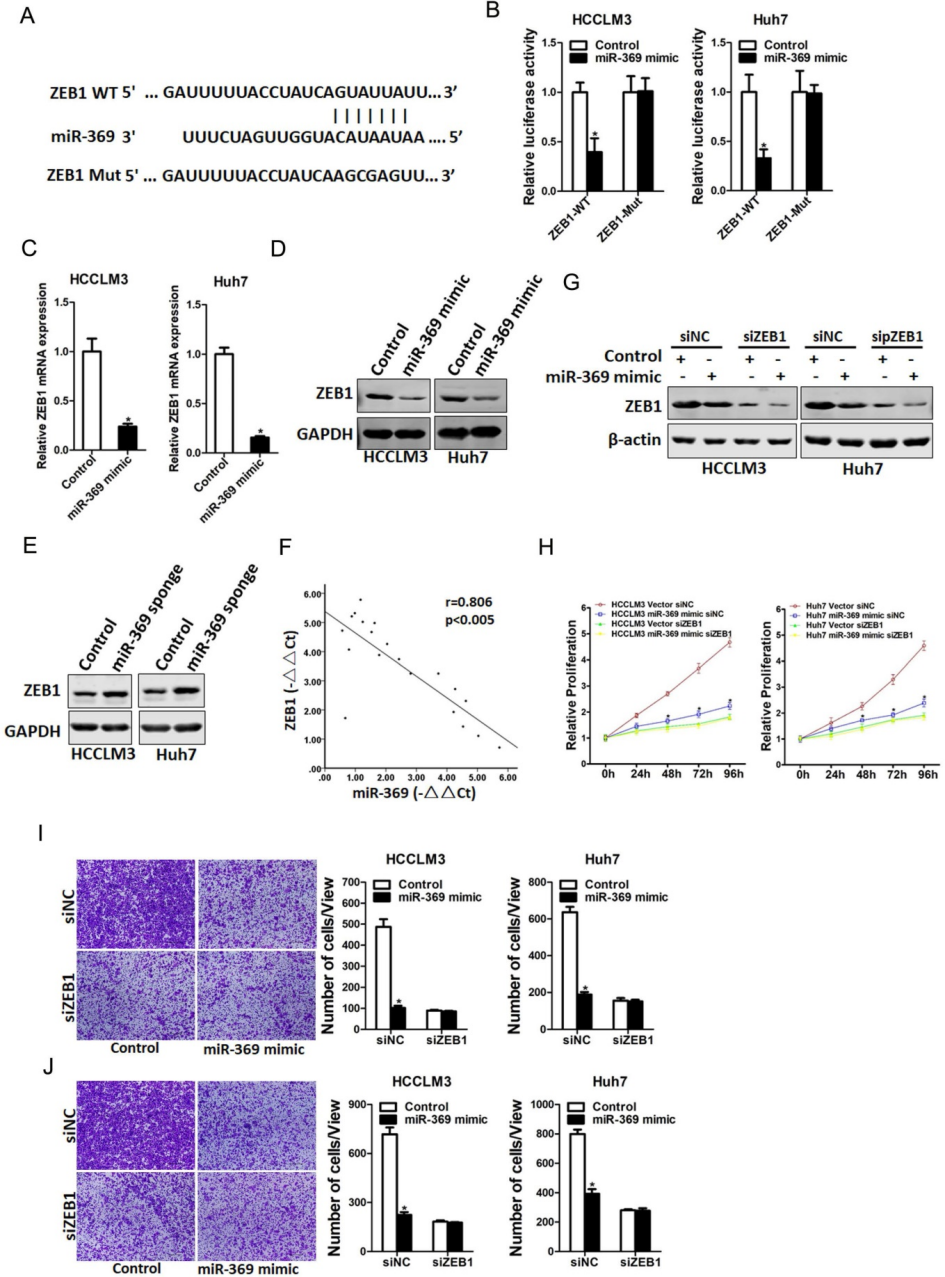
** ZEB1 is a direct target of miR-369 in HCC cells. A.** A potential target site for miR-369 in the 3'-UTR of human ZEB1 mRNA, as predicted by the program Targetscan. To disrupt the interaction between miR-369 and ZEB1 mRNA, the target site was mutated. **B.** Luciferase reporter assays performed in HCCLM3 miR-369 mimic or Huh7 miR-369 mimic and their control cells transfected with wild-type or mutant ZEB1 3'-UTR constructs. **C.** The mRNA expression of ZEB1 was checked in HCCLM3 miR-369 mimic or Huh7 miR-369 mimic and their control cells by real-time PCR. **D.** The protein expression of ZEB1 was checked in HCCLM3 miR-369 mimic or Huh7 miR-369 mimic and their control cells by western blot. GAPDH was used as an invariant control. **E.** The protein expression of ZEB1 was checked in HCCLM3 miR-369 sponge or Huh7 miR-369 sponge and their control cells by western blot. GAPDH was used as an invariant control. **F.** Significant correlation was observed between miR-369 and ZEB1 expression in human HCC tissues (n=20). **G.** HCCLM3 miR-369 mimic or Huh7 miR-369 mimic and their control cells were transfected with ZEB1 siRNA and then checked by western bolt assay. GAPDH was used as an invariant control. **H.** HCCLM3 miR-369 mimic or Huh7 miR-369 mimic and their control cells were transfected ZEB1 siRNA and then subjected to CCK8 assay. **I.** HCCLM3 miR-369 mimic or Huh7 miR-369 mimic and their control cells were transfected ZEB1 siRNA and then subjected to migration assay. **J.** HCCLM3 miR-369 mimic or Huh7 miR-369 mimic and their control cells were transfected ZEB1 siRNA and then subjected to Invasion assay.

Next, we attempted to identify the potential target genes of miR-369 in HCC cells. ZEB1 was predicted by TargetScan to be a candidate targeted gene in human and mouse for miR‐369, a conserved miRNA complementarily pairing the 3′‐UTR sequences of ZEB1 (Fig. 5A). To further explore whether miR-369 directly regulates ZEB1 expression via interaction with its 3'-UTR, the wild-type or mutant ZEB1 3'-UTR reporter plasmids were transfected into miR-369 mimic HCC cells and their control cells. The luciferase activity of wild-type reporter was significantly downregulated in miR-369 overexpression HCC cells (Fig. 5B). However, miR-369-mediated repression of the reporter expression was abolished by mutation of the miR-369 binding site in the ZEB1 3'-UTR. Moreover, ZEB1 mRNA and protein expression was also decreased in miR-369 overexpression HCC cells (Fig. 5C&D). Conversely, ZEB1 protein expression was increased in miR-369 knockdown HCC cells (Fig. 5E). Furthermore, there was a significant negative correlation between miR-369 and ZEB1 mRNA expression in human HCC tissues (Fig. 5F).

To further investigate the role of ZEB1 in miR-369-mediated proliferation and metastasis of HCC cells, special ZEB1 siRNA was transfected into miR-369 overexpression HCC cells and control cells (Fig. 5G). As shown in Fig. 5G, ZEB1 siRNA abrogated the distinct growth capacity between miR-369 overexpression HCC cells and control cells (Fig. 5H). Consistently, ZEB1 siRNA also abolished the discrepancy of metastasis between miR-369 overexpression cells and their control cells (Fig. 5I&J), which further confirmed that miR369 inhibited HCC cells proliferation and metastasis by directly target ZEB1.

## Discussion

Numerous studies showed that miRNAs are discovered in human cancers and played important roles in genes regulation at post-transcriptional levels 21, 22. But there are still many unknown miRNAs. So, it's urgent to explore new functional miRNAs in human cancers. Previous studies demonstrated that miR-369 was involved in numerous tumors regulation 23-25. In this study, we for first found that the expression of miR-369 was significantly downregulated in human liver fibrosis and liver cancer tissues. The analysis of patient cohort demonstrated that miR-369 predicted the poor prognosis of HCC patients. Overexpressing of miR-369 inhibited HCC cells proliferation and metastasis by targeting ZEB1.

It was reported that miRNAs participated in regulation of HCC 26, 27. For instance, miR‐369 was downregulated in papillary thyroid cancer and inhibited papillary thyroid cancer cells proliferation by downregulating TSPAN13 15. Therefore, we hypothesize that miR-369 could also regulate HCC cells proliferation. In the present study, we confirmed that overexpression of miR-369 decreased the HCC cells proliferation and tumor growth by a series of *in vitro* and *in vivo* assays. Through the CCK8 and colony formation analyses, we found that miR-369 could suppress HCC cells *in vitro*. Further animal experiments analyses demonstrated that overexpression of miR-369 could inhibit HCC cells *in vivo*, which was consistent with previous research results.

Additionally, we also found that overexpression of miR-369 attenuated the migration and invasive ability of HCC cell lines *in vitro* and *in vivo*, indicating that miR-369 might be involved in tumor metastasis, which happens very early in HCC patients. Metastasis a process that cancer cells attenuate cell-cell adhesion and disseminate into distant organs, in which epithelial-mesenchymal transition (EMT) plays a critical role 28. EMT is an orchestrated series of events, in which epithelial cells lose their properties and acquire mesenchymal phenotypes, resulting in a loss of epithelial polarity and reduced intercellular adhesion 29, 30. Recently, an increasing study have reported that miRNAs family members including miR-369 induce EMT and aggressiveness in human cancer 31, 32. Consistently, our study revealed that overexpression of miR-369 induced EMT by increasing the expression of epithelial marker E-cadherin and reducing the expression of mesenchymal marker vimentin and N-cadherin. These findings indicated that miR-369 might play a role in tumor metastasis.

Zinc finger E-box binding homeobox 1 (ZEB1) is a crucial member of the zinc finger-homeodomain transcription factor family, originally identified as a binding protein of the lens-specific δ1-crystalline enhancer and is a pivotal transcription factor in the epithelial-mesenchymal transition (EMT) process 33. ZEB1 also plays a vital role in embryonic development and cancer progression, including breast cancer progression 34, 35. However, the exact mechanism beneath ZEB1 activation in HCC remains vague. We hereby revealed that ZEB1 is a direct target of miR-369 in HCC cells. miR-369 mimic decreased ZEB1 mRNA and protein expression in HCC cells. Moreover, we also found that miR-369 directly regulates ZEB1 expression via interaction with its 3'-UTR. More importantly, ZEB1 siRNA could abrogated growth capacity or metastasis ability between miR-369 mimic cells and control HCC cells. Herein, we for first revealed that miR-369 inhibited HCC cells proliferation and metastasis via directly regulating ZEB1. These findings of the present study not only shed a new light on the mechanism of HCC but suggest a potential therapeutic target against HCC patients.

In conclusion, our study investigated for the first time the biological significance of miR-369 in HCC. We have demonstrated that the expression level of miR-369 was reduced in human liver fibrosis and liver cancer tissues. The analysis of patient cohort demonstrated that miR-369 predicted the poor prognosis of HCC patients. Overexpression of miR-369 inhibited the HCC cells proliferation and metastasis. Moreover, the tumor-inhibiting effect of miR-369 may be partially related the inactivation of ZEB1. Therefore, miR-369 might serve as a potential therapeutic target for HCC patients.

## Supplementary Material

Supplementary figures and tables.
