# Supplementary material for: miR-369 inhibits Liver Cancer progression by targeting ZEB1 pathway and predicts the prognosis of HCC patients
Source: J Cancer. 2021 Mar 19;12(10):3067–76. doi: 10.7150/jca.54759 (PMC8040887; doi:10.7150/jca.54759)
Supplement: Supplementary file 1 — Supplementary figures and tables. [file jcav12p3067s1.pdf]

miR-369 Inhibits Liver Cancer Progression by targeting ZEB1 Pathway and Predicts the Prognosis of HCC Patients

Yuwei Dong[1, #], Fuxia Li[2, #], Junjun Wang[1, #], Jiangfeng Hu[1], Zhenghong Li[1], Yubei Gu[3,*], Yun Feng[1,*]

[1]Department of Gastroenterology, Shanghai General Hospital, School of Medicine, Shanghai Jiao Tong University. Shanghai, 200080, China.
[2]Department of General Surgery, Cao County People's Hospital, Heze, Shandong province, 274400, China.
[3]Department of Gastroenterology, Rui Jin Hospital, School of Medicine, Shanghai Jiao Tong University. Shanghai, 200025, China.

# These authors contribute equally to this paper and are the co-first authors of this paper.
Corresponding authors: Department of Gastroenterology, Shanghai General Hospital, School of Medicine, Shanghai Jiao Tong University. No 100, Hai Ning Road, Shanghai, 200080, China. Email address: yun.feng2@shgh.cn (Yun Feng); gyb11809@rjh.com.cn (Yubei Gu).

Running title: miR-369 inhibits HCC cells expansion.

Funding: This work was supported by grants from the National Natural Science Foundation of China: 81900544; sponsored by shanghai sailing program: 19YF1439000.

Competing interests: All authors declare no competing interests.

Supplementary Figure 1

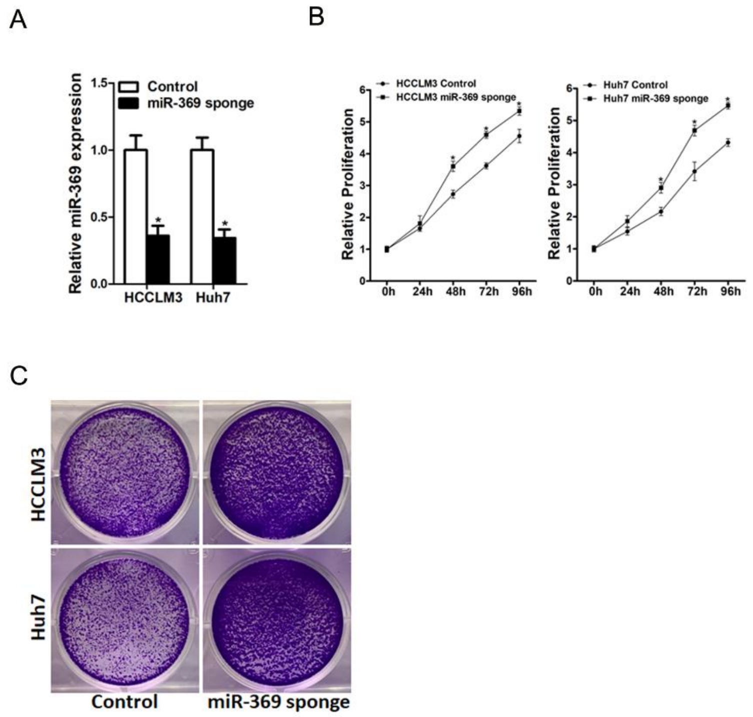

Supplementary Figure 1

A. The knockdown effect of miR-369 in HCCLM3 and Huh7 cells was checked by real-time PCR assay.

B. Cell proliferation was measured using CCK-8 assays in HCCLM3 miR-369 sponge or Huh7 miR-369 sponge and their control cells.

C. Colony formation assays of HCCLM3 miR-369 sponge or Huh7 miR-369 sponge and their control cells.

Supplementary Figure 2

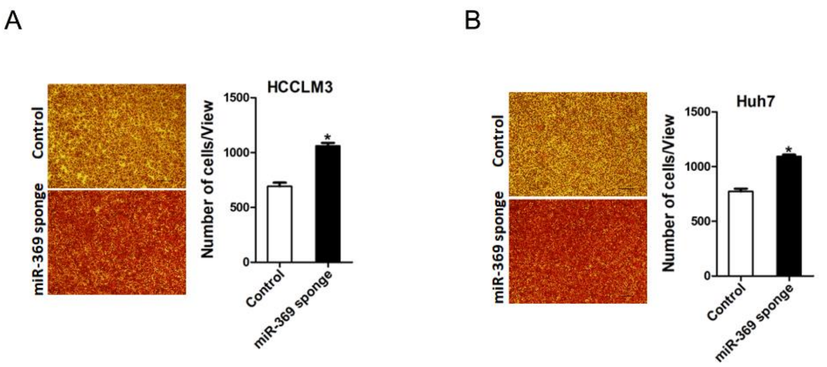

Supplementary Figure 2

A. The invasion ability of HCCLM3 miR-369 sponge and its control cells was performed utilizing polycarbonate membrane inserts in a 24-well plate.
B. The invasion ability of Huh7 miR-369 sponge and its control cells was performed utilizing polycarbonate membrane inserts in a 24-well plate.

Supplementary Tables

Supplementary Table 1. Clinicopathological features of 120 HCC patients in Cohort 1

Characteristics		miR-369 low (n=60)	miR-369 high (n=60)	p value
Age(year)	≤50	36	37	>0.05
	>50	24	23	
Gender	Male	48	47	>0.05
	Female	12	13	
HBsAg	Positive	45	31	<0.05
	Negative	15	29	
AFP(μg/L)	≤400	10	28	<0.05
	>400	50	32	
Tumor size(cm)	≤5	14	28	<0.05
	>5	46	32	
Tumor number	Single	35	37	>0.05
	Multiple	25	23	
Portal vein tumor thrombus	Yes	25	10	<0.05
	No	35	50	
Pathological satellite	Yes	30	32	>0.05

	No	30	28	
BCLC stage	A	21	34	<0.05
	B or C	39	26	
TNM	I-II	46	45	>0.05
	III-IV	14	15	

HBsAg: hepatitis B virus surface antigen; AFP: α-fetoprotein; TNM: Tumor-Nodes-Metastasis; BCLC: Barcelona Clinic Liver Cancer Staging.
